# D2 dopamine receptor gene (*DRD2*) Taq1A (*rs1800497*) affects bone density

**DOI:** 10.1038/s41598-020-70262-0

**Published:** 2020-08-06

**Authors:** Ting-I. Chiang, Hsien-Yuan Lane, Chieh-Hsin Lin

**Affiliations:** 1 Department of Psychiatry, Kaohsiung Chang Gung Memorial Hospital, Chang Gung University College of Medicine, No. 123, Dapi Rd., Niaosong District, Kaohsiung City, 833 Taiwan; 2 Graduate Institute of Biomedical Sciences, China Medical University, No. 91, Hsueh-Shih Rd., North Dist., Taichung City, 404 Taiwan; 3 Department of Psychiatry and Brain Disease Research Center, China Medical University Hospital, Taichung, Taiwan; 4 Department of Psychology, College of Medical and Health Sciences, Asia University, Taichung, Taiwan; 5 School of Medicine, Chang Gung University, Taoyuan, Taiwan

**Keywords:** Clinical genetics, Predictive markers, Genetics research

## Abstract

Schizophrenia patients are susceptible to lower bone mineral density (BMD). However, studies exploring the genetic effects are lacking. Genes that affect the activity of antipsychotics may be associated with BMD, particularly in patients receiving long-term antipsychotic treatment. We aimed to explore the relationship between the dopamine receptor D_2_ (*DRD2*) gene Taq1A (*rs1800497*) polymorphism and BMD in chronic schizophrenia patients. We recruited schizophrenia patients (n = 47) and healthy controls (n = 39) from a medical center in Taiwan and collected data that may affect BMD. Patients’ BMD was measured by dual-energy X-ray absorptiometer (DEXA). *DRD2 rs1800497* was genotyped through polymerase chain reaction–Restriction Fragment Length Polymorphism (PCR–RFLP). Among all participants, subjects with *DRD2 rs1800497*(T;T) allele had lower DEXA T score and DEXA Z score compared to those with *rs1800497*(C;T) and *rs1800497*(C;C) alleles (*p* = 0.008, 0.003, respectively). In schizophrenia patients, subjects with *rs1800497*(T;T) allele also had lower DEXA Z score compared to the other two alleles (*p* = 0.045). Our findings suggest that individuals with the *DRD2 rs1800497*(T;T) had lower BMD than those with the *rs1800497*(C;T) and *rs1800497*(C;C) genotypes. Therefore, genes should be considered as one of the risk factors of lower BMD.

## Introduction

With an increasingly ageing society, decreased bone mineral density (BMD) is an issue worthy of more attention. The operational description of osteoporosis is a BMD value at the femoral neck of 2.5 standard deviations (SDs) or more below the young female adult mean (T-score less than or equal to − 2.5 SDs)^1^. The World Health Organization (WHO) describes it as a “progressive systemic skeletal disease characterized by low bone mass and microarchitectural deterioration of bone tissue, with a consequent increase in bone fragility and susceptibility to fracture”^2^. People suffering from osteoporosis are at an increased risk for bone fracture, thus also leading to disability, diminished quality of life, and mortality. Europe has for the greatest number of osteoporotic fractures (34.8%), which lead to the loss of more Disability Adjusted Life Years (DALYs) than common cancers, with the exception of lung cancer^3^. In Asia, the average treatment cost of osteoporosis-related hip fracture is around US $2,943, which is roughly 18.95% of the countries' 2014 GDP/capita^4^.

Schizophrenia is among the most critical psychiatric diseases in the world. These patients have markedly premature mortality and a variety of physical comorbidities. Schizophrenia patients are at a greater risk for diabetes, chronic obstructive pulmonary disease (COPD), and influenza or pneumonia^5^. Recent studies have indicated that schizophrenia is associated with reduced BMD and higher fracture risk^6,7^. Hyperprolactinemia (HPRL) is a common side effect of schizophrenia patients taking such antipsychotics as first-generation antipsychotics (FGA) and certain second-generation antipsychotics (SGA). Some studies suggest a relationship between antipsychotic-induced hyperprolactinemia (HPRL) and BMD loss^6,8^. HPRL may contribute to BMD loss through two potential mechanisms. The direct mechanism is through the pathway of osteoblast cells, while the indirect one is through the suppression of gonadal hormone levels via the hypothalamic–pituitary–gonadal axis^9^. The etiology of both low bone mineral density (LBMD) and osteoporosis is multi-faceted, and lifestyle, psychotic symptoms, medication use, alcohol consumption, and genes may all be related^10,11^. In addition to acquired factors that influence BMD, congenital factors like gene polymorphism also potentially play an important role.

Dopamine is the predominant neurotransmitter in the central nervous system involved in a wide variety of physiological processes^12^. Dopamine has five different seven-transmembrane G protein-coupled receptors named D1 to D5^13^. The D2 receptor is mainly located in the striatum, olfactory tubercle and the core of nucleus accumbens^14^. It is known that D2 receptor is related to motor activity, reinforcement mechanisms, as well as learning and memory^13^. Some studies found that dopamine receptor D2 (*DRD2*) gene was involved in genetic susceptibility to posttraumatic stress disorder, risk factors associated with smoking and schizophrenia^15–17^. The *DRD2 rs1800497* polymorphisms were found to be related with body weight, pathological eating behavior and risk of alcoholism^18,19^. In the genetic aspects, several studies have suggested that individuals with *DRD2 rs1800497* T allele have a higher plasma prolactin level under antipsychotic use^20–22^.

So far, few studies have explored possible genetic effects on BMD. The latent relationship between the *DRD2 rs1800497* allele and BMD in schizophrenia patients remains unclear. Therefore, in this study, we aimed to clarify the relationship between *DRD2 rs1800497* gene and bone mineral density.

## Methods

### Setting

This study was performed in the outpatient department and chronic inpatient units of Kaohsiung Chang Gung Memorial Hospital, a major psychiatric center in south Taiwan. The hospital offers inpatients a balanced diet that contains 600 mg calcium and approximately 2000 cal/day. The study was approved by the Institutional Review Board of Kaohsiung Chang Gung Memorial Hospital, and all participants provided their written informed consent in accordance with the Declaration of Helsinki after being provided a complete description of the study.

### Patients

We enrolled patients diagnosed with chronic schizophrenia by research psychiatrists based on the DSM-IV criteria (American Psychiatric Association, 1994). All patients had been clinically ill but stable in psychotic symptoms. They had been treated with unchanged antipsychotics and doses for at least 6 months. According to the antipsychotic regimens at the time of enrollment, we separated patients into two groups: patients taking first-generation antipsychotics, risperidone, paliperidone, amisulpride, or ziprasidone were defined as the prolactin-releasing (PR) group; while those taking clozapine, olanzapine, quetiapine, or aripiprazole were defined as the prolactin-sparing (PS) group. The various antipsychotic medications were converted and expressed as a chlorpromazine equivalent dose (mg/day)^23^. Such information as education duration, age of disease onset, disease duration, hospitalization duration, and antipsychotic treatment duration was also recorded. All participants were Han Chinese in Taiwan.

### Controls

We also enrolled 39 unrelated healthy volunteers from the community and staff of Kaohsiung Chang Gung Memorial Hospital. Ages ranged from 18 to 65 years old, and all participants were Han Chinese in Taiwan. Healthy volunteers were free from any axis I psychiatric disorder.

### Exclusion criteria

Both patients and healthy individuals with the following physical or mental conditions that may have influenced BMD were excluded: eating disorder, substance abuse/dependence (including smoking and alcohol drinking, which are forbidden in hospitals and public places in Taiwan), renal function impairment, electrolyte imbalance, bone metabolism diseases, thyroid or parathyroid diseases, pituitary tumor, pregnancy or lactation, and co-medications known to influence BMD (e.g., glucocorticoids^24^, heparin^25^, and drugs for osteoporosis like parathyroid hormones^26^, alendronate^27^, selective estrogen receptor modulators^28^, and bisphosphonates, estrogens, and calcitonin^29^, with the exception of benzodiazepines and antidepressants, which have not been significantly associated with reduced BMD^8^).

### Assessments

We used measurements from the dual-energy X-ray absorptiometer (DEXA)^10,23,24^ to assess bone marrow density at lumbar spine L2–L4 in a supine position. Professional radiologists, who were all blinded to the patients’ clinical characteristics, evaluated these data.

An absolute BMD value T score between − 2.5 and − 1 was defined as osteopenia^25^, while a T score of − 2.5 or lower was defined as osteoporosis^26^. A T score of − 1 or less was considered LBMD (including osteopenia and osteoporosis)^25^. We also measured DEXA Z score to evaluate whether decreasing bone mineral density originated from aging^27^. A Z score of − 1 or less was considered bone mineral loss with causes except age^27^.

Blood samples were collected at 08:00 to assess bone remodeling-related factors, including complete blood and platelet count, serum estradiol, testosterone, LH, FSH, prolactin, cortisol, thyroid hormone, TSH, Free-T4, T3, blood urea nitrogen, creatinine, alkaline phosphatase, and calcium. Data from physical examinations, including body weight, height, and BMI, were also recorded.

We analyzed *DRD2 rs1800497* genotype by polymerase chain reaction-Restriction Fragment Length Polymorphism (PCR–RFLP). First, we designed a suitable sense primer and antisense primer to amplify the candidate gene (primers sequence, *rs1800497*_F: CCGTCGACGGCTGGCCAAGTTGTCTA; *rs1800497*_R: CCGTCGACCCTTCCTGAGTGTCATCA). The total volume for PCR was 30 μl, including 1 μl DNA template, 3 μl of ten times PCR buffer, 1.5 μl DMSO, 0.75 μl dNTP (5 mM/μl), 0.45 μl sense primer (10 pmol/μl), 0.45 μl antisense primer (10 pmol/μl), 0.45 μl FastStar Taq DNA polymerase (5 U/μl), and 21.9 μl ddH2O. We used MJ PTC-200 for amplification. Reaction criteria for PCR were 95 °C for 5 min, 35 cycles of 95 °C for 30 s, 61 °C for 30 s, 72 °C for 30 s, and the last cycle of 72 °C for 7 min. We used 2% agarose gel to confirm the product, then cut it using a suitable limited-enzyme. The post-cut PCR product was then submitted to electrophoresis by 3% high resolution agarose and dyed with ethidium bromide to obtain the final genotype result. *DRD2 Taq1A* A1A1 referred to *rs1800497*(T;T), *DRD2 Taq1A* A2A2 referred to *rs1800497*(C;C), and *DRD2 Taq1A* A1A2 referred to *rs1800497*(C;T).

Research psychiatrists collected patients’ clinical data, including history of psychiatric and physical illness and medication kind and duration, by reviewing medical records and through structured clinical interviews. The Positive and Negative Syndrome Scale (PANSS) (Kay et al. 1987; Buchanan et al. 2005) and the Global Assessment of Functioning (GAF) (axis V of DSM-IV) were used to evaluate the severity of psychopathological symptoms and overall functioning. An intraclass correlation coefficient of 0.95 was obtained among the raters.

### Statistical analysis

We adopted the chi-squared test or Fisher’s exact test as appropriate for between-group comparisons of categorical data. We used the t-test to analyze continuous data. Analysis of Variance (ANOVA) was used to determine the relationship between *DRD2 rs1800497* genotype and BMD. We also performed post hoc analysis to assess the difference between *rs1800497* genotypes. All tests were two-tailed, and statistical significance of tests was defined as *p* < 0.05. Data were analyzed with SPSS version 22.0 (SPSS Inc., USA).

## Results

### Patients and controls

This study consisted of a total of 47 patients and 39 controls. Participants’ demographic and clinical characteristics are shown in Table 1. Age, free T4, TSH, renal function (BUN, creatinine), and BMD (DEXA T score, DEXA Z score) were similar between patients and controls. The schizophrenia group had more men than women compared to the control group. Patients with schizophrenia had significantly higher levels of BMI, T3, ALK-P, prolactin, and FSH than the controls. The mean estradiol level of schizophrenia patients was significantly lower than that of controls.

**Table 1:** Demographic and clinical characteristics of schizophrenia patient group and control group.

Characteristics	Controls (N = 39)		Patients (N = 47)		Test statistic	*p*
	Mean	SD	Mean	SD		
Gender (M/F)	11/28		27/20			**0.009**^†^
Age (years)	39.46	8.13	41.88	10.04	− 1.212	0.254
Education duration (years)	15.41	1.48	12.26	1.75	8.912	0.709
Disease onset age (years)			25.33	9.37		
Duration of disease (months)			203.46	107.97		
Duration of admission (days)			871.73	1,446.38		
Duration of antipsychotic use (days)			5,871.96	3,155.60		
Chlorpromazine equivalence dose (mg/day)			420.49	219.63		
PANSS (total score)			90.32	21.28		
PANSS-positive score			21.21	6.87		
PANSS-negative score			21.57	4.96		
PANSS-general score			47.32	11.65		
Global Assessment of Functioning score			54.94	9.07		
Body weight (kg)	60.41	11.86	75.05	14.81	− 4.985	0.250
Height (cm)	163.39	7.54	164.98	8.83	− 0.890	0.240
BMI	22.48	3.21	27.48	4.55	− 5.775	**0.017**
Cortisol (μg/dl)	10.64	4.98	13.11	4.54	− 2.423	0.773
Free T4 (μg/dl)	0.87	0.16	0.81	0.17	1.798	0.076
T3 (ng/dl)	95.18	14.90	99.55	19.33	− 1.159	**0.037**
TSH (mIU/l)	1.69	1.14	1.64	1.31	0.197	0.975
Calcium (mg/dl)	9.09	0.35	8.95	0.34	1.901	0.794
Alkaline phosphatase (U/l)	49.95	15.20	70.60	18.57	− 5.588	**0.035**
BUN (mg/dL)	12.54	3.42	11.01	3.14	2.174	0.474
Creatinine (mg/dl)	0.72	0.17	0.81	0.17	− 2.556	0.778
Osteocalcin (ng/ml)	14.62	5.32	18.31	7.39	− 2.615	0.096
Prolactin (ng/ml)	11.59	6.88	20.59	23.80	− 2.281	**< 0.001**
FSH (mIU/ml)	8.86	11.65	15.95	23.40	− 1.724	**0.020**
Estradiol (ng/ml)	93.49	82.57	40.03	46.31	3.811	**0.001**
LH (mIU/ml)	10.18	15.40	9.91	10.98	0.095	0.312
Testosterone (ng/ml)	1.45	1.90	2.57	2.09	− 2.594	0.240
DEXAT	0.05	1.26	− 0.0021	1.38	0.195	0.843
DEXAZ	0.28	1.13	0.34	1.11	− 0.242	0.731
BMDSCORE	1.02	0.15	1.03	0.16	− 0.166	0.821

*PANSS* Positive and Negative Syndrome Scale.

^†^Fisher’s exact test.

### Genotype and bone mineral density

**Figure 1:**
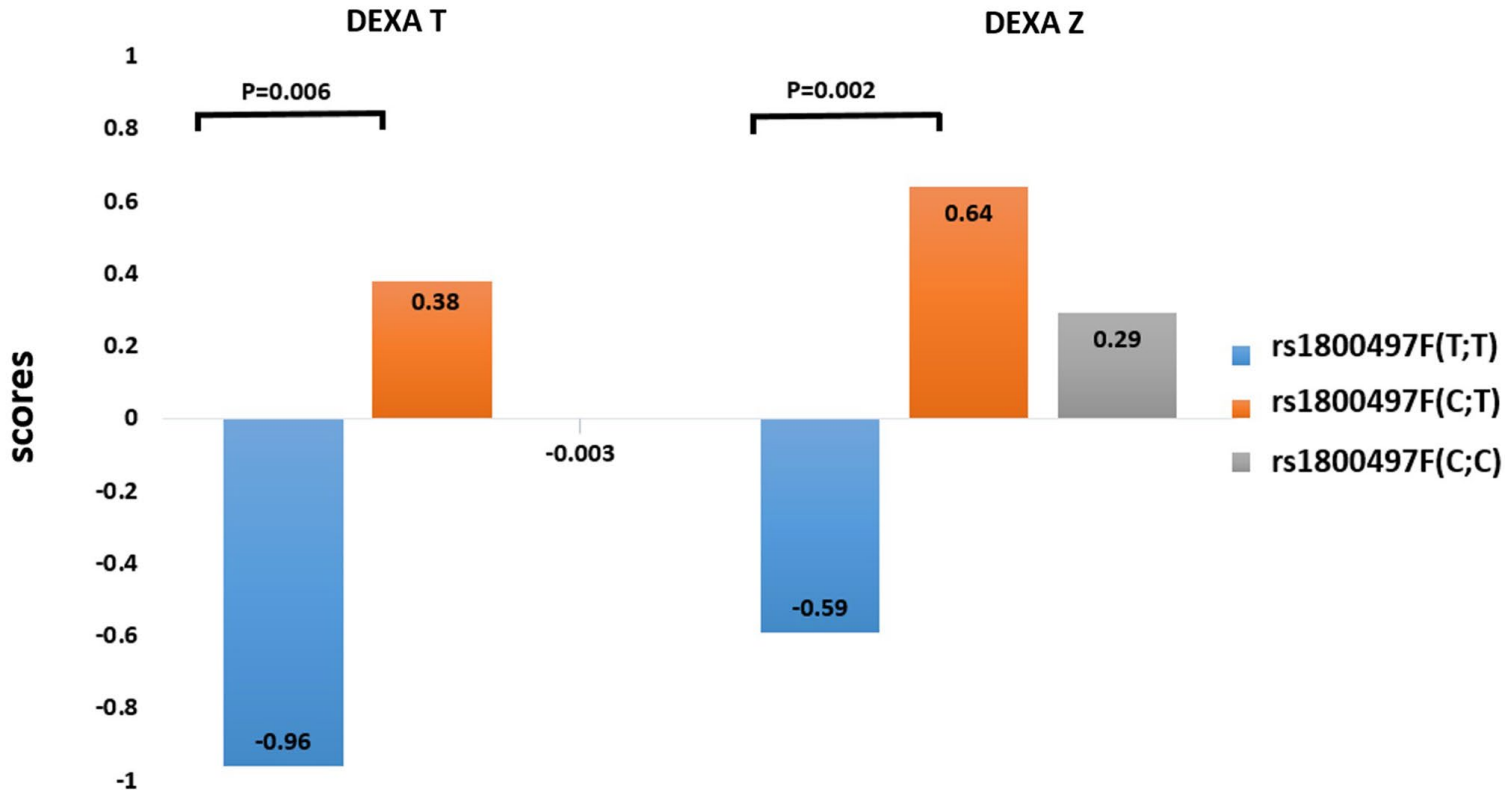
For all participants (both schizophrenia patients and controls), different DEXAT (*p* = 0.008) and DEXAZ (*p* = 0.003) scores were found among different *DRD2 rs1800497* genotypes. Post hoc analysis revealed lower bone density in the (T;T) allele than in the (C;T) allele in DEXAT (*p* = 0.006) and DEXAZ (*p* = 0.002) scores.

The relationships between *DRD2 rs1800497* genotype and BMD (including DEXA T, DEXA Z, and BMD scores) were analyzed by ANOVA and Bonferroni correction (Table 2). For all participants (both schizophrenia patients and controls), the mean bone densities were different among different *rs1800497* genotypes (*p* = 0.008, 0.003 and 0.014 for DEXA T, DEXA Z and BMD scores, respectively (Fig. 1). Post hoc analysis with Bonferroni correction revealed lower bone density in the *rs1800497*(T;T) allele group than in the (C;T) allele group in DEXA T (*p* = 0.006), DEXA Z (*p* = 0.002), and BMD (*p* = 0.011) scores. We observed no significant differences in bone density between (T;T) allele versus (C;C) allele and (C;T) allele versus (C;C) allele (Table 2). Notably, the aforementioned tests did not reach statistical significance when applying the multiple testing corrections.

**Table 2:** Analysis of Variance (ANOVA) of *DRD2 rs1800497* genotype and bone density in all participants.

Bone mineral density measurement	*DRD2 rs1800497* genotype	N	Mean	SD	*p* value^a^	Post hoc analysis	
							*p* value
DEXAT	(T;T)	12	− 0.96	1.12	**0.008**	(T;T)/(C;C)	0.073
	(C;C)	38	− 0.003	1.36		(T;T)/(C;T)	**0.006**
	(C;T)	36	0.38	1.18		(C;C)/(C;T)	0.591
	Total	86	0.02	1.32			
DEXAZ	(T;T)	12	− 0.59	1.05	**0.003**	(T;T)/(C;C)	0.042
	(C;C)	38	0.29	1.07		(T;T)/(C;T)	**0.002**
	(C;T)	36	0.64	1.04		(C;C)/(C;T)	0.465
	Total	86	0.31	1.12			
BMD score	(T;T)	12	0.92	0.12	**0.014**	(T;T)/(C;C)	0.102
	(C;C)	38	1.02	0.16		(T;T)/(C;T)	**0.011**
	(C;T)	36	1.06	0.14		(C;C)/(C;T)	0.676
	Total	86	1.02	0.15			

^a^Post hoc analysis with Bonferroni correction.

In schizophrenia patients, the *rs1800497*(T;T) group had lower DEXA Z scores (*p* = 0.045) than the *rs1800497*(C;C) and (C;T) groups (Table 3). The *rs1800497*(T;T) group also had lower DEXA T scores (*p* = 0.070) and BMD scores (*p* = 0.075) than the other two genotypes. In the controls, *rs1800497*(T;T) group had lower DEXA Z scores (*p* = 0.076), DEXA T scores (*p* = 0.054), and BMD scores (*p* = 0.125) compared to the *rs1800497* (C;C) and (C;T) groups.

**Table 3:** Analysis of variance (ANOVA) of *DRD2 rs1800497* genotype and bone mineral density measurement in schizophrenia patients and controls.

			Patients	Controls
DEXAT	(T;T)	N	5	7
		Mean (SD)	− 1.02 (1.21)	− 0.91 (1.14)
	(C;C)	N	21	17
		Mean (SD)	− 0.20 (1.40)	0.24 (1.32)
	(C;T)	N	21	15
		Mean (SD)	0.43 (1.27)	0.30 (1.08)
	Total	N	47	39
		Mean (SD)	− 0.002 (1.38)	0.05 (1.26)
	*p* value		0.070	0.076
DEXAZ	(T;T)	N	5	7
		Mean (SD)	− 0.52 (1.09)	− 0.64 (1.11)
	(C;C)	N	21	17
		Mean (SD)	0.16 (1.04)	0.45 (1.11)
	(C;T)	N	21	15
		Mean (SD)	0.72 (1.08)	0.52 (1.01)
	Total	N	47	39
		Mean (SD)	0.34 (1.11)	0.28 (1.13)
	*p* value		**0.045**	0.054
	Post hoc analysis	(T;T)/(C;C)	0.621	0.090
		(T;T)/(C;T)	0.070	0.070
		(C;C)/(C;T)	0.273	1.000
BMD score	(T;T)	N	5	7
		Mean (SD)	0.91 (0.14)	0.92 (0.13)
	(C;C)	N	21	17
		Mean (SD)	1.00 (0.16)	1.04 (0.16)
	(C;T)	N	21	15
		Mean (SD)	1.08 (0.15)	1.04 (0.13)
	Total	N	47	39
		Mean (SD)	1.03 (0.16)	1.02 (0.15)
	*p* value		0.075	0.125

We also analyzed the relationship between different *rs1800497* genotypes and prolactin level in all individuals. As shown in Table 4, after adjusting gender, we found that prolactin levels were different among PR group, PS group, and controls in the *rs1800497* (T;T) genotype (*p* = 0.000023) and the (C;T) genotype (*p* = 0.000003), respectively. In the *rs1800497*(T;T) group, post hoc analysis with Bonferroni correction showed higher prolactin level in PR group comparing to PS group and controls (*p* = 0.000059, 0.000027, respectively). Similarly, in the *rs1800497*(C;T) group, post hoc analysis with Bonferroni correction showed higher prolactin level in PR group comparing to PS group and controls (*p* = 0.000029, 0.000011, respectively). In the *rs1800497*(C;C) group, there was no significant difference in prolactin level among subgroups. We further examined the effect of *rs1800497* genotypes, gender and prolactin level on DEXAZ score using multiple linear regression model. We found that *rs1800497* was associated with DEXAZ score while adjusting gender and prolactin level (*p* = 0.001) (Table 5).

**Table 4:** Analysis of variance (ANOVA) of prolactin level (ng/ml) and DEZAZ score among *DRD2 rs1800497* genotypes in all individuals (schizophrenia patients and controls).

*DRD2 rs1800497* genotype		PR patients	PS patients	Controls	*p* value	*p* value of post hoc analysis
(T;T)	N	2	3	7	0.000023	
	ProL Mean ± SD	44.0 ± 5.9	5.9 ± 5.0	7.2 ± 3.9		PR versus PS (*p* = 0.000059) PR versus controls (*p* = 0.000027)
	DEXAZ mean ± SD	− 0.9 ± 1.6	− 0.3 ± 1.0	− 0.6 ± 1.1	0.873	
(C;C)	N	6	15	17		
	ProL mean ± SD	29.8 ± 33.1	15.8 ± 22.9	12.3 ± 8.9	0.129	
	DEXAZ mean ± SD	− 0.4 ± 0.8	0.4 ± 1.1	0.5 ± 1.1	0.265	
(C;T)	N	10	11	15	0.000003	
	ProL mean ± SD	34.7 ± 27.7	9.1 ± 5.5	12.9 ± 4.5		PR versus PS (*p* = 0.000029) PR versus controls (*p* = 0.000011)
	DEXAZ mean ± SD	0.4 ± 1.0	1.0 ± 1.1	0.5 ± 1.0	0.402	
Total	N	18	29	39		
	ProL mean ± SD	34.7 ± 27.4	12.1 ± 16.8	11.6 ± 6.9	0.000007	PR versus PS (*p* = 0.000044) PR versus controls (*p* = 0.000012)
	DEXAZ mean ± SD	− 0.0 ± 1.0	0.5 ± 1.1	0.3 ± 1.1	0.242	

The analyses were adjusted for gender.

PR, prolactin-raising antipsychotics; PS, prolactin-sparing antipsychotics; ProL, prolactin level.

**Table 5:** Multiple linear regression analysis (stepwise) of independent factors associated with DEXAZ score.

Variable	*B* (SE)	*t*	*p* value
*DRD2 rs1800497*	0.563 (0.163)	3.448	0.001
Gender	0.199 (0.232)	0.859	0.393
Prolactin level	− 0.009 (0.006)	− 1.476	0.144
Adjusted *R*^2^ = 0.123			

To test the potential confounders that influence the genetic effect on BMD, variables including gender, BMI, T3, alkaline phosphatase, estradiol, and prolactin, which significantly differed between schizophrenia patients and controls, were adjusted in the analysis of covariance. The DEXAZ scores were still different between the *rs1800497*(T;T) genotype and (C;T) genotype (*p* = 0.049) after adjusting the aforementioned variables. Regarding DEXAT scores and BMD scores, the differences in bone density between the *rs1800497*(T;T) genotype and (C;T) genotype did not reach statistical significance (0.055 and 0.055, respectively) (Supplementary Table S1).

## Discussion

In this study, we found that participants with the *DRD2 rs1800497*(T;T) genotype had lower bone density than those with (C;C) or (C;T) genotypes. The trend remained similar after adjusting potential covariates. The findings suggest that individuals with *DRD2 rs1800497*(T;T) genotype might be prone to have lower bone density than the other two genotypes. To the best of our knowledge, this study is the first to demonstrate the possible relationship between *DRD2 rs1800497* polymorphism and bone density.

There was not yet definite mechanism for the relationship between *DRD2 rs1800497* and BMD so far. However, several recent studies found that *DRD2* had impact on bone homeostasis. Hanami K et al. found that dopamine decreased osteoclast differentiation via D2-like receptor expressed on human osteoclast precursor cells^30^. Besides, dopamine D2-like receptor signaling can inhibit osteoclastogenesis and bone resorption in vitro^30^. In mice study, administration of D2 receptor antagonist amisulpride to diabetic mice restored trabecular bone structure to near normal and partially reversed downregulation of lysyl oxidase^31^. Moreover, bromocriptine, a potent D2 agonist has been widely used in the treatment of bone metastatic prostate cancer^32^. Several studies have found that people with the *DRD2 rs1800497* T allele had a higher prolactin level during antipsychotics use^20^. A recent meta-analysis of patients with schizophrenia showed that prolactin levels were significantly higher in *rs1800497* T carriers than T non-carriers^21^. Previous studies explored the relationship between antipsychotics-induced hyperprolactinemia and BMD loss, which have resulted in inconsistent findings^33^. Many factors contribute to the regulation of prolactin level, including environment, physiological stimuli, sexual hormones, medication, etc.^34^. Whether *DRD2 rs1800497* controls bone density through elevated prolactin level or other mechanisms unrelated to hyperprolactinemia requires further investigation. A larger-scale study is warranted to clarify the relationship between *DRD2 rs1800497* and prolactin level.

*DRD2 rs1800497* polymorphism is associated with various psychiatric diseases. Substance use disorders, such as cocaine, nicotine, opioid, and particularly alcohol dependence, were related to *DRD2*^35^. Large-scale meta-analysis has confirmed that *DRD2 rs1800497* polymorphism was associated with alcohol dependence and even HIV positive alcohol abusers^36,37^. Furthermore, individuals with the *rs1800497*(C;C) allele have a higher likelihood of smoking cessation than those who carry the (T;T) or (C;T) allele^38^. In more recent studies, *rs1800497* polymorphism was demonstrated in post-traumatic brain injury cognitive performance and Parkinson disease^39,40^.

As increasing research attempts to explore BMD via genetic-level and widespread genome-wide association studies (GWAS), several genes have been found to be associated with bone density. The key genes related to bone fragility include LDL receptor-related protein 5 (LRP5), Wnt Family Member 1 (WNT1), and Plastin 3 (PLS3)^41^. A recent systematic review and meta-analysis study demonstrated significant correlations between vitamin D receptor (VDR) polymorphisms, such as VDR ApaI and VDR FokI, and susceptibility to postmenopausal osteoporosis^42^. Such studies have offered us the opportunity to understand the bone density issue at the genetic level and increased our knowledge about osteoporosis^43^. A GWAS study integrating co-expression network data found that *SPTBN1* (spectrin, beta, non-erythrocytic 1) and *MARK3* (MAP/microtubule affinity-regulating kinase 3) were causal genes at BMD GWAS loci^44^. In addition to GWAS, genome-scale capture C based method found that novel implicated genes such as *ING3* and *EPDR1* were related to osteoblastogenesis^45^. A large-scale GWAS study revealed DEXA bone area associated genes, including *GDF5* (*rs143384*), *COL11A1* (*rs3753841*) and microRNA *MIR196A2* gene (*rs11614913*[T])^46^. To our knowledge, there is not yet publically available GWAS data investigating *DRD2 rs1800497* polymorphism and bone density. So far, this research is the first to explore the possible relationship between *DRD2 rs1800497* polymorphism and bone density.

Our study has several limitations. First, the etiology of BMD loss is multifactorial, including age, gender, daily activities, sun exposure and others. Genetic effect is only one of many contributors. Furthermore, we only investigated one candidate gene in this study rather than using a large scale population-based GWAS study which may provide stronger evidence. Second, the sample size was relatively small. Under multiple testing corrections, the *p* values would not reach statistical significance since there were multiple tests. Third, such risk factors as diet, exercise, and sun exposure that may affect BMD were difficult to control strictly, particularly for outpatients, although we provided adequate education for every participant. Fourth, although we arranged physical and laboratory evaluations to exclude conditions that may affect BMD as much as possible, we were unable to completely exclude all physical conditions. Fifth, this study was a cross-sectional study. Further longitudinal studies are warranted to confirm our finding. Lastly, only Han Chinese participants were enrolled in this study. Further research with other ethnicities is required.

In summary, this study revealed for the first time that individuals with the *DRD2 rs1800497*(T;T) allele had lower bone density when compared to (C;C) and (C;T) alleles. Physicians should consider genetic effect when assessing the risk of osteoporosis or osteopenia. Future studies are required to elucidate the underlying mechanisms of *DRD2 rs1800497*′s influence on bone density.

## Supplementary information

Supplementary information.

## Data Availability

The datasets used and/or analyzed during the current study are available from the corresponding author on reasonable request.

## References

[1] Kanis JA, McCloskey EV, Harvey NC, Johansson H, Leslie WD (2015). Intervention thresholds and the diagnosis of osteoporosis. J. Bone Miner. Res..

[2] Kanis JA, Melton LJ, Christiansen C, Johnston CC, Khaltaev N (1994). The diagnosis of osteoporosis. J. Bone Miner. Res..

[3] Johnell O, Kanis JA (2006). An estimate of the worldwide prevalence and disability associated with osteoporotic fractures. Osteoporos. Int..

[4] Mohd-Tahir NA, Li SC (2017). Economic burden of osteoporosis-related hip fracture in Asia: A systematic review. Osteoporo. Int..

[5] Crump C, Winkleby MA, Sundquist K, Sundquist J (2013). Comorbidities and mortality in persons with schizophrenia: A Swedish national cohort study. Am. J. Psychiatry.

[6] Kishimoto T, De Hert M, Carlson HE, Manu P, Correll CU (2012). Osteoporosis and fracture risk in people with schizophrenia. Curr. Opin. Psychiatry.

[7] Stubbs B (2015). Schizophrenia and the risk of fractures: A systematic review and comparative meta-analysis. Gen. Hosp. Psychiatry.

[8] Kinjo M, Setoguchi S, Schneeweiss S, Solomon DH (2005). Bone mineral density in subjects using central nervous system-active medications. Am. J. Med..

[9] Graham SM (2011). Risk of osteoporosis and fracture incidence in patients on antipsychotic medication. Expert Opin. Drug Saf..

[10] Javaid MK, Holt RI (2008). Understanding osteoporosis. J. Psychopharmacol..

[11] Lu S (2017). Bivariate genome-wide association analyses identified genetic pleiotropic effects for bone mineral density and alcohol drinking in Caucasians. J. Bone Miner. Metab..

[12] Bjorklund A, Dunnett SB (2007). Dopamine neuron systems in the brain: An update. Trends Neurosci..

[13] Missale C, Nash SR, Robinson SW, Jaber M, Caron MG (1998). Dopamine receptors: From structure to function. Physiol. Rev..

[14] Bouthenet M-L (1991). Localization of dopamine D3 receptor mRNA in the rat brain using in situ hybridization histochemistry: Comparison with dopamine D2 receptor mRNA. Brain Res..

[15] Comings DE, Muhleman D, Gysin R (1996). Dopamine D2 receptor (DRD2) gene and susceptibility to posttraumatic stress disorder: A study and replication. Biol. Psychiatry.

[16] Dubertret C (2004). The 3′ region of the DRD2 gene is involved in genetic susceptibility to schizophrenia. Schizophr. Res..

[17] Comings DE (1996). The dopamine D2 receptor (DRD2) gene: A genetic risk factor in smoking. Pharmacogenetics.

[18] Munafo MR, Matheson IJ, Flint J (2007). Association of the DRD2 gene Taq1A polymorphism and alcoholism: A meta-analysis of case–control studies and evidence of publication bias. Mol. Psychiatry.

[19] Nisoli E (2007). D2 dopamine receptor (DRD2) gene Taq1A polymorphism and the eating-related psychological traits in eating disorders (anorexia nervosa and bulimia) and obesity. Eat. Weight Disord..

[20] Calarge CA (2009). Variants of the dopamine D2 receptor gene and risperidone-induced hyperprolactinemia in children and adolescents. Pharmacogenet. Genomics.

[21] Miura I (2016). Variants in the DRD2 locus and antipsychotic-related prolactin levels: A meta-analysis. Psychoneuroendocrinology.

[22] Aklillu E (2007). CYP2D6 and DRD2 genes differentially impact pharmacodynamic sensitivity and time course of prolactin response to perphenazine. Pharmacogenet. Genomics.

[23] Gardner DM, Murphy AL, O'Donnell H, Centorrino F, Baldessarini RJ (2010). International consensus study of antipsychotic dosing. Am. J. Psychiatry.

[24] Suzuki Y, Sato S (2010). Secondary osteoporosis UPDATE. Clinical significance of glucocorticoid-induced osteoporosis. Clin. Calcium.

[25] Wawrzynska L, Tomkowski WZ, Przedlacki J, Hajduk B, Torbicki A (2003). Changes in bone density during long-term administration of low-molecular-weight heparins or acenocoumarol for secondary prophylaxis of venous thromboembolism. Pathophysiol. Haemost. Thromb..

[26] Rosen CJ (2005). The role of parathyroid hormone in the management of osteoporosis. Horm. Res..

[27] Iwamoto J, Sato Y, Uzawa M, Takeda T, Matsumoto H (2010). Seven years' experience with alendronate in postmenopausal Japanese women with osteoporosis. Therap. Clin. Risk Manag..

[28] Kulak Junior J, Kulak CA, Taylor HS (2010). SERMs in the prevention and treatment of postmenopausal osteoporosis: An update. Arq. Bras. Endocrinol. Metabol..

[29] Bakker SC (2007). The PIP5K2A and RGS4 genes are differentially associated with deficit and non-deficit schizophrenia. Genes Brain Behav..

[30] Hanami K (2013). Dopamine D2-like receptor signaling suppresses human osteoclastogenesis. Bone.

[31] Daley EJ, Pajevic PD, Roy S, Trackman PC (2019). Impaired gastric hormone regulation of osteoblasts and lysyl oxidase drives bone disease in diabetes mellitus. JBMR Plus.

[32] Yang Y (2018). Repositioning dopamine D2 receptor agonist bromocriptine to enhance docetaxel chemotherapy and treat bone metastatic prostate cancer. Mol Cancer Ther..

[33] Peuskens J, Pani L, Detraux J, De Hert M (2014). The effects of novel and newly approved antipsychotics on serum prolactin levels: A comprehensive review. CNS Drugs.

[34] Freeman ME, Kanyicska B, Lerant A, Nagy G (2000). Prolactin: Structure, function, and regulation of secretion. Physiol. Rev..

[35] Noble EP (2000). Addiction and its reward process through polymorphisms of the D2 dopamine receptor gene: A review. Eur. Psychiatry.

[36] Wang F, Simen A, Arias A, Lu QW, Zhang H (2013). A large-scale meta-analysis of the association between the ANKK1/Rs1800497 polymorphism and alcohol dependence. Hum. Genet..

[37] Agudelo M (2014). Alcohol abuse and HIV infection: Role of DRD2. Curr. HIV Res..

[38] Ma Y, Wang M, Yuan W, Su K, Li MD (2015). The significant association of Taq1A genotypes in DRD2/ANKK1 with smoking cessation in a large-scale meta-analysis of Caucasian populations. Transl. Psychiatry.

[39] Failla MD (2015). Posttraumatic brain injury cognitive performance is moderated by variation within ANKK1 and DRD2 genes. J. Head Trauma Rehabil..

[40] McDonell KE, van Wouwe NC, Harrison MB, Wylie SA, Claassen DO (2018). Taq1A polymorphism and medication effects on inhibitory action control in Parkinson disease. Brain Behav..

[41] Rocha-Braz MG, Ferraz-de-Souza B (2016). Genetics of osteoporosis: Searching for candidate genes for bone fragility. Arch. Endocrinol. Metab..

[42] Zhang L (2018). Associations between VDR gene polymorphisms and osteoporosis risk and bone mineral density in postmenopausal women: A systematic review and meta-analysis. Sci. Rep..

[43] Boudin E, Van Hul W (2017). Mechanisms in endocrinology: Genetics of human bone formation. Eur. J. Endocrinol..

[44] Calabrese GM (2017). Integrating GWAS and co-expression network data identifies bone mineral density genes SPTBN1 and MARK3 and an osteoblast functional module. Cell Syst..

[45] Chesi A (2019). Genome-scale capture C promoter interactions implicate effector genes at GWAS loci for bone mineral density. Nat. Commun..

[46] Styrkarsdottir U (2019). GWAS of bone size yields twelve loci that also affect height, BMD, osteoarthritis or fractures. Nat. Commun..

