# Supplementary material for: D2 dopamine receptor gene (DRD2) Taq1A (rs1800497) affects bone density
Source: Sci Rep. 2020 Aug 6;10:13236. doi: 10.1038/s41598-020-70262-0 (PMC7414035; doi:10.1038/s41598-020-70262-0)
Supplement: Supplementary file 1 — Supplementary information. [file 41598_2020_70262_MOESM1_ESM.docx]

**D2 dopamine Receptor Gene (*DRD2*) Taq1A (*rs1800497*) Affects Bone Density**

**Ting-I Chiang, M.D. ^a^, Hsien-Yuan Lane,** **M.D., Ph.D.^b, c, d,*^, Chieh-Hsin Lin, M.D., Ph.D. ^a, b, e, *^**

^a^ Department of Psychiatry, Kaohsiung Chang Gung Memorial Hospital, Chang Gung University College of Medicine, Kaohsiung, Taiwan

^b^ Graduate Institute of Biomedical Sciences, China Medical University, Taichung, Taiwan

^c^ Department of Psychiatry & Brain Disease Research Center, China Medical University Hospital, Taichung, Taiwan

^d^ Department of Psychology, College of Medical and Health Sciences, Asia University, Taichung, Taiwan

^e^ School of Medicine, Chang Gung University, Taoyuan, Taiwan

Address correspondence to co-corresponding author *

Hsien-Yuan Lane, M.D., Ph.D., Graduate Institute of Biomedical Sciences, China Medical University, No. 91, Hsueh-Shih Rd., North Dist., Taichung City 404, Taiwan; e-mail address: hylane@gmail.com

Chieh-Hsin Lin, M.D., Ph.D., Department of Psychiatry, Kaohsiung Chang Gung Memorial Hospital, No. 123, Dapi Rd., Niaosong District, Kaohsiung City 833, Taiwan; e-mail address: cyndi36@gmail.com

Running Title: *DRD2* affects bone density

**Supplementary Information**

| **Supplementary Table S1.** Analysis of covariance (ANCOVA) of *DRD2 rs1800497* genotype and bone mineral density measurement in schizophrenia patients and controls | | | | |
| --- | --- | --- | --- | --- |
| Bone mineral density measurement | *DRD2 rs1800497*  genotype | B | SE | P value |
| DEXAT | (T;T)/ (C;C) | -1.081 | 0.682 | 0.124 |
|  | (T;T)/ (C;T) | -1.560 | 0.779 | **0.055** |
|  | (C;C)/ (C;T) | -0.479 | 0.582 | 0.372 |
| DEXAZ | (T;T)/ (C;C) | -0.939 | 0.576 | 0.113 |
|  | (T;T)/ (C;T) | -1.353 | 0.658 | **0.049** |
|  | (C;C)/ (C;T) | -0.414 | 0.446 | 0.361 |
| BMDSCORE | (T;T)/ (C;C) | -0.139 | 0.083 | 0.104 |
|  | (T;T)/ (C;T) | -0.190 | 0.095 | **0.055** |
|  | (C;C)/ (C;T) | -0-050 | 0.064 | 0.441 |
